# Comparative transcriptomic analysis for identification of candidate sex-related genes and pathways in Crimson seabream (*Parargyrops edita*)

**DOI:** 10.1038/s41598-020-80282-5

**Published:** 2021-01-13

**Authors:** Binbin Shan, Yan Liu, Changping Yang, Yu Zhao, Dianrong Sun

**Affiliations:** 1 Key Laboratory of South China Sea Fishery Resources Exploitation & Utilization, Ministry of Agriculture Rural Affairs, Guangzhou, China; 2 Guangdong Provincial Key Laboratory of Fishery Ecology and Environment, Guangzhou, China; 3 South China Sea Fisheries Research Institute, Chinese Academy of Fisheries Sciences, Guangzhou, China

**Keywords:** Marine biology, Gene expression

## Abstract

Teleost fishes display the largest array of sex-determining systems among animals, resulting in various reproductive strategies. Research on sex-related genes in teleosts will broaden our understanding of the process, and provide important insight into the plasticity of the sex determination process in vertebrates in general. Crimson seabream (*Parargyrops edita* Tanaka, 1916) is one of the most valuable and abundant fish resources throughout Asia. However, little genomic information on *P. edita* is available. In the present study, the transcriptomes of male and female *P. edita* were sequenced with RNA-seq technology. A total of 388,683,472 reads were generated from the libraries. After filtering and assembling, a total of 79,775 non redundant unigenes were obtained with an N50 of 2,921 bp. The unigenes were annotated with multiple public databases, including NT (53,556, 67.13%), NR (54,092, 67.81%), Swiss-Prot (45,265, 56.74%), KOG (41,274, 51.74%), KEGG (46,302, 58.04%), and GO (11,056, 13.86%) databases. Comparison of the unigenes of different sexes of *P. edita* revealed that 11,676 unigenes (9,335 in females, 2,341 in males) were differentially expressed between males and females. Of these, 5,463 were specifically expressed in females, and 1,134 were specifically expressed in males. In addition, the expression levels of ten unigenes were confirmed to validate the transcriptomic data by qRT-PCR. Moreover, 34,473 simple sequence repeats (SSRs) were identified in SSR-containing sequences, and 50 loci were randomly selected for primer development. Of these, 36 loci were successfully amplified, and 19 loci were polymorphic. Finally, our comparative analysis identified many sex-related genes (*zps, amh, gsdf, sox4, cyp19a*, etc.) and pathways (MAPK signaling pathway, p53 signaling pathway, etc.) of *P. edita*. This informative transcriptomic analysis provides valuable data to increase genomic resources of *P. edita*. The results will be useful for clarifying the molecular mechanism of sex determination and for future functional analyses of sex-associated genes.

## Introduction

Sex determination is the process that establishes the sex of an individual and regulates the differentiation of sex characteristics, and its mechanisms are highly conserved in many animals (e.g. mammals and birds)^1,2^. Many of the genes involved in sex differentiation in distantly related species are the same, suggesting that the gene regulation mechanisms underlying sex differentiation and sex determination might be similar^3,4^. However, the mechanisms of sex determination in teleost fishes are remarkably diverse^5,6^. Among animals, teleost fishes showed the most diverse systems of sex determination, resulting in numerous reproductive strategies^7,8^. In contrast to sex in birds, humans and other higher vertebrates, which is relatively stable, sex in teleost fishes is unstable or alterable^9–11^. Research on sex determination mechanisms in teleosts has aroused widespread interest in an increasing number of investigators. It will provide deep insight into the plasticity of the sex determination process and broaden understanding of the process. In addition, as teleosts are an important protein resource for humans, it is important to understand the reproductive biology of teleosts to enable prediction of potential impacts and effective management.

Crimson seabream (*Parargyrops edita* Tanaka, 1916), a demersal fish belonging to the family Sparidae, is naturally distributed from the southern coast of Japan to the coast of Indonesia^12^. *P*. *edita* is one of the most valuable and popular fish resources throughout Asia, but catches dropped from ca. 49,000 t in 1970 to 21,000 t in 2008 due to overfishing in the Beibu Gulf^13^. With high overfishing, *P*. *edita* populations have shown miniaturization, prematurity and structural change. Given the important economic status and population decline of the species, many studies have been performed to access the stock and to examine the population genetic variation and biology of *P*. *edita*^12,14^. However, the genomic resources of *P*. *edita* are insufficient. Only mitochondrial gene sequences in the National Center for Biotechnology Information (NCBI) database can be used. Therefore, screening genes related to sex and identifying the functions of these genes may not only enable characterization of the mechanisms of the gene network underlying sex determination and differentiation in *P*. *edita*, but also enlarge the genomic database for *P*. *edita* and support future molecular biology studies.

Benefiting from the rapid development of next generation sequencing (NGS) technologies, high-throughput RNA sequencing (RNA-seq) is becoming readily available for non-model organisms^15,16^. The transcriptome contains almost all protein-coding genes, representing an essential part of the genome^17^. For species whose genomes are still not available or that have complex genomes, sequencing the transcriptome is an attractive option for detecting genes, especially in economically valuable fish. In addition, transcriptomic analysis enables simultaneous analyses of multiple processes, including protein homeostasis, metabolism and other regulatory cellular processes^8,18,19^. Recently, transcriptomics has been applied to identify sex-determining genes in teleost fishes^20–22^. In the present study, RNA-seq analysis was performed on pooled samples of multiple tissues from *P*. *edita* of different sexes. In addition, expression profiles were generated to detect differentially expressed genes (DEGs) between male and female *P*. *edita*. Subsequently, the candidate sex-related genes were determined. Furthermore, abundant simple sequence repeats (SSRs) were detected among deep-coverage sequence region reads. The results will provide a foundation for discovering regulatory mechanisms associated with sex-related genes and a valuable transcriptomic resource, which will facilitate research on the molecular regulatory mechanisms of teleost fishes.

## Materials and methods

### Ethics statement

All the experimental procedures were approved by the ethics committee of Laboratory Animal Welfare and Ethics of South China Sea Fisheries Research Institute (project identification code: nhdf 2019-02, date of approval: 13th Oct. 2019). The methods involving animals in this study were conducted in accordance with the Laboratory Animal Management Principles of China.

### Fish materials

Six mature *P. edita* (three females and three males) were collected from the Beibu Gulf of China (N 19°56.552, E 108°02.841). To eliminate confounding effects, the *P. edita* were temporarily acclimated for three days in an aerated seawater tank. Then, all fish were anaesthetized and killed by severing the spinal cord. Then, tissues (muscle, gonad, liver and heart tissues) from each *P. edita* specimen were collected and frozen in liquid nitrogen for total RNA extraction.

### RNA extraction, cDNA library building, and sequencing

Total RNA was extracted from each tissue of the six fish using TRIzol reagent. RNA integrity was assessed by using an Agilent 2100 Bioanalyzer (Agilent Technologies, Palo Alto, CA, USA), and the samples with RNA integrity numbers (RINs) ≥ 7 were subjected to subsequent analysis^23^. RNA from tissues from every individual was pooled in equal amounts.

Then, mRNA (RNA with a poly A tail) was extracted from the total RNA using magnetic beads with Oligo (dT) probes and purified. Fragmentation buffer was applied to break the mRNA into fragments of suitable size, and the fragmented mRNA was reverse-transcribed into double-stranded cDNA with the N6 random primer. Then, the cDNA fragments were repaired with phosphate at the 5′end, and an “A” base was added to the 3′ end, after which adapters were ligated to the cDNA fragments. PCR was performed to amplify the ligation products. After heat denaturation, the single-stranded DNA was cyclized with a splint oligo and DNA ligase. Finally, the prepared cDNA libraries were sequenced on the BGISEQ-500 platform using a 150-bp paired-end strategy (BGI, Shenzhen, China; http://bgitechsolutions.com/technologies/64).

### De novo assembly and functional annotation

Trimmomatic was used to remove adaptors as well as low-quality sequences from the raw reads generated from the BGISEQ-500 platform^24^. First, the parameter in ILLUMINACLIP was set to 2:30:10 to remove the adaptor, leading and trailing bases with a Phred quality score below 3 were trimmed, reads were scanned with a 4 bp sliding window and trimmed when average Phred quality dropped below 20, and reads with a length lower than 50 bp were also discarded. Subsequently, the Trinity software package (trinityrnaseq, version: 2.0.6; https://github.com/trinityrnaseq/trinityrnaseq/releases/tag/v2.0.6) was applied to de novo assembly of the high-quality clean reads (min_kmer_cov: 3)^25^. The k-mer value was set to 25. TGICL clustering software (version: 2.1) was then used to remove the redundancies and acquire maximum-length nonredundant unigenes with a minimum of 70% similarity^26^. Benchmarking Universal Single-Copy Orthologs (BUSCO) was then used to evaluate transcriptome completeness, using a < 15% missing genes in the BUSCO Eukaryota dataset as a benchmark for analysis^27^. Finally, all unigenes were annotated by comparison against different public databases, including the NCBI non-redundant protein (NR) database, the NCBI nucleotide (NT) database, the Swiss-Prot protein database, the Gene Ontology (GO) databases, the Kyoto Encyclopedia of Genes and Genomes (KEGG) database and the euKaryotic Orthologous Group (KOG) database (BLASTx 2.5.0; the E-value threshold was 1 × 10^−5^).

### Analysis of potential sex-related genes

In order to characterize gene expression variation in the samples from different sexes, we used the short-read alignment tool Bowtie2 to map all clean reads to the assembled transcriptome, with end-to-end alignment, a 22-base seed length, two reseed attempts with no mismatches allowed in the seed, a seed interval of 17 bases, and all other settings at the default values ^28^. To determine the fragments per kilobase of exon model per million mapped fragments (FPKM) values, RSEM was applied to normalize the expression levels of all unigenes^29^. To identify DEGs, the DEGseq package was applied^30^. The threshold for DEGs was defined as an absolute log_2_(fold change) value ≥ 4 and a Q-value ≤ 0.001. Among the DEGs, unigenes specifically expressed in female or male crimson seabream were regarded as specifically expressed genes (SEGs). Furthermore, enrichment analyses (KEGG and GO) of the DEGs were performed based on the hypergeometric distribution test, and a significance test was applied. A *P* value ≤ 0.05 indicated a significantly enriched term.

### Potential SSR marker detection and validation

The MIcroSAtellite identification tool (MISA, version 1.0) was applied to identify the simple sequence repeat (SSR) sequences in the assembled transcriptome. The minimum repeat number for the various unit types were as follows: twelve for mono-nucleotide, six for dinucleotide, five for tri-nucleotide and tetra-nucleotide, and four for penta-nucleotide and hexa-nucleotide microsatellites. Then, primers for every SSR locus were designed on flanking sequences by using Primer 3^31^.

In order to assess the polymorphism of the SSR markers, we used a wild *P. edita* population sampled in the Beibu Gulf, genomic DNA was isolated from 24 randomly selected *P. edita* specimens using a marine animal DNA kit (Tiangen, Beijing, China). Then, 1% agarose gel electrophoresis was used to access the integrity of the DNA samples. Fifty pairs of primers were initially selected and tested. The annealing temperatures of the primers were initially tested for amplification using pooled DNA samples. PCR amplifications were carried out using a Mastercycler gradient thermal cycler (Eppendorf) in a final volume of 25 μl, and the mixture and conditions of PCR followed the methods of Shan et al^32^. Subsequently, genomic DNA from 24 samples were amplificated under appropriate annealing temperatures, and the products were resolved in 6% denaturing polyacrylamide gels and visualized by silver staining.

### Validation of the transcriptome data by quantitative real-time PCR

To validate the accuracy of the crimson seabream transcriptomic data, ten genes were selected for quantitative real-time PCR (qRT-PCR) validation. All the selected genes exhibited significantly different expression between female samples and male samples; there were five female-biased genes and five male-biased genes. Primer design was carried out using Primer Premier 6.0 (PREMIER Biosoft International, Palo Alto, California). In addition, 18 s and β-actin were chosen as reference genes for internal standardization, and the PCR procedure and amplification reaction system were the same as those described by Lou et al^16^. ABI7300 SDS software (Applied Biosystems) was applied to analyze the data after the PCR program was run. We calculated the relative expression levels of the ten target unigenes based on the 2^−ΔΔCt^ method (ΔCT = CT_target unigene_ − CT_reference gene_, − ΔΔCT = ΔCT_female_ − ΔCT_male_) and the log_2_(fold change) was then used for a comparison with the log_2_(fold change) of RNA-seq.

## Results

### Transcriptome sequencing and de novo assembly

Six cDNA libraries were constructed and subsequently sequenced on the BGISEQ-500 platform. In total, 388,683,472 reads were generated, and 373,965,788 (96.21%) clean reads were screened after removal of adaptor sequences, N-containing sequences and low-quality sequences. The statistics of the six cDNA libraries are shown in Table 1. All clean reads from the females and males were assembled to generate unigenes (Table 1). After clustering and removal of redundancies with Trinity and TGICL, 79,775 unigenes were generated with an N50 length of 2,921 bp and a mean length of 1,523 bp (Table 2).

**Table 1:** Summary statistics for the transcriptome sequencing data from each sample.

Sample	Raw reads (M)	Clean reads (M)	Clean bases (Gb)	Clean reads Q30 (%)	Clean reads Ratio (%)
Male-1	64.75	62.73	6.27	88.77	96.89
Male-2	65.26	62.73	6.27	88.13	96.12
Male-3	65.87	62.74	6.27	87.65	95.25
Female-1	64.75	62.80	6.28	87.69	96.99
Female-2	63.45	61.27	6.13	88.64	96.57
Female-3	64.61	61.68	6.17	88.03	95.48

**Table 2:** Statistics for the assembled unigenes.

Sample	All unigenes	Male-1	Male-2	Male-3	Female-1	Female-2	Female-3
Total number	79,775	59,199	55,106	64,955	53,326	48,526	47,929
Total length	121,550,499	67,647,958	58,700,462	66,129,252	68,587,072	54,148,211	56,319,295
Mean length	1,523	1,142	1,065	1,018	1,286	1,115	1,175
N50	2,921	2,177	2,022	1,930	2,446	2,158	2,208
N70	1,887	1,280	1,146	1,078	1,499	1,233	1,341
N90	649	427	395	372	501	411	448
GC (%)	46.69	46.68	46.66	46.88	46.68	46.51	47.29

The raw reads of the present study were uploaded to the SRA databases of NCBI under BioProject PRJNA591405, with accession numbers SRR10568165-SRR10568170. This Transcriptome Shotgun Assembly project has been deposited at DDBJ/EMBL/GenBank under the accession number GICI00000000. The version described in this paper is the first version, GICI01000000.

### Functional annotation of the *P. edita* transcriptome

**Figure 1:**
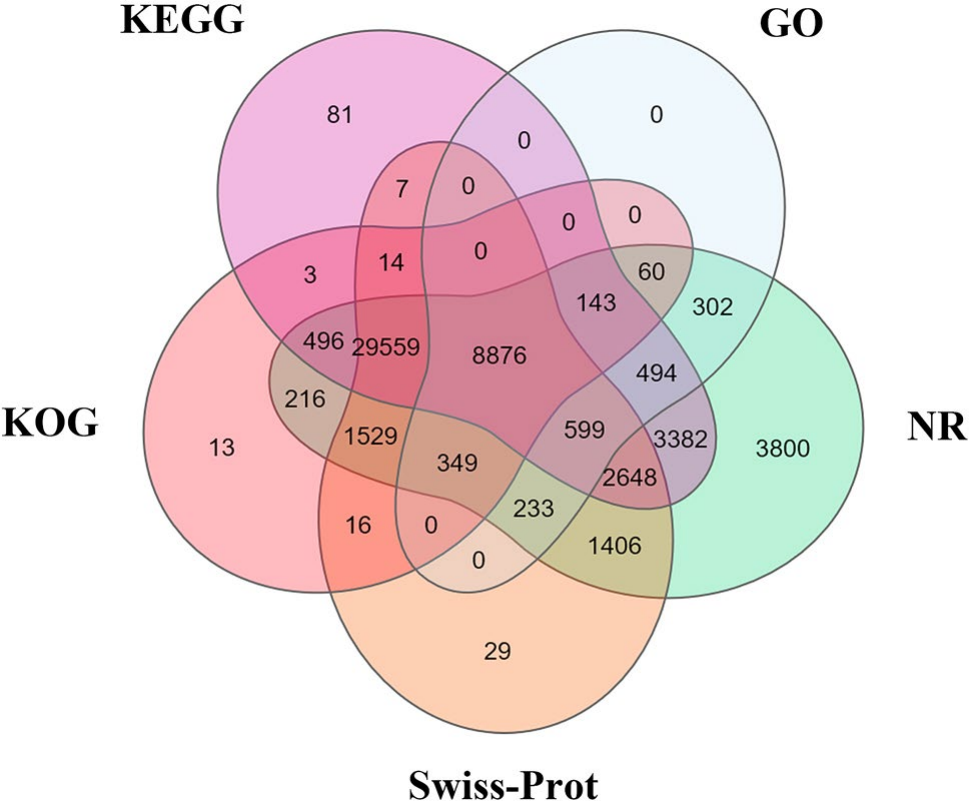
Venn diagram of the functional annotation results.

All the unigenes were functionally characterized based on the descriptions of their similar sequences from databases. The numbers and percentages of unigenes that matched to various databases are shown in Table 3. In summary, 53,556 unigenes (67.13%) were annotated to the NCBI nucleotide NT database A total of 54,255 unigenes (68.01%) were annotated to at least one of the protein databases, while 8,876 unigenes (11.13%) were annotated to all the protein databases that were searched in this study, including the NR, Swiss-Prot, KEGG, KOG and GO databases (Fig. 1). Subsequently, the completeness of the assembly and annotations was assessed with BUSCO. The results showed that 99% of the unigenes (51% as single genes and 48% as duplicated genes) were found to be complete, and 1% were found to be fragmented (Figure S1).

**Table 3:** Summary of unigene annotations.

Database	Number of unigenes	Percentage (%)
Total	79,775	100.00
NT	53,556	67.13
NR	54,092	67.81
Swiss-Prot	45,265	56.74
KEGG	46,302	58.04
KOG	41,274	51.74
GO	11,056	13.86

**Figure 2:**
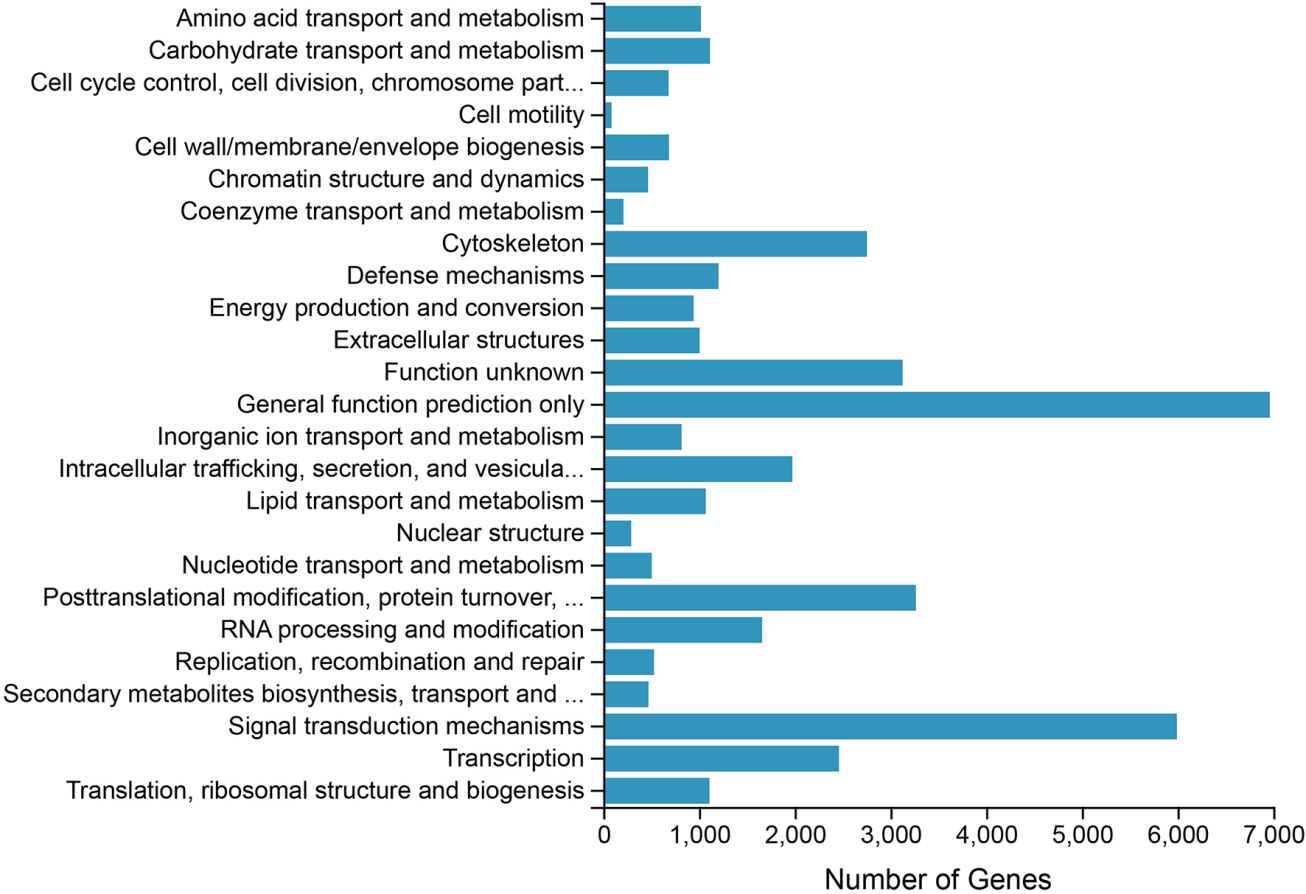
Clusters of KOG functional classifications.

**Figure 3:**
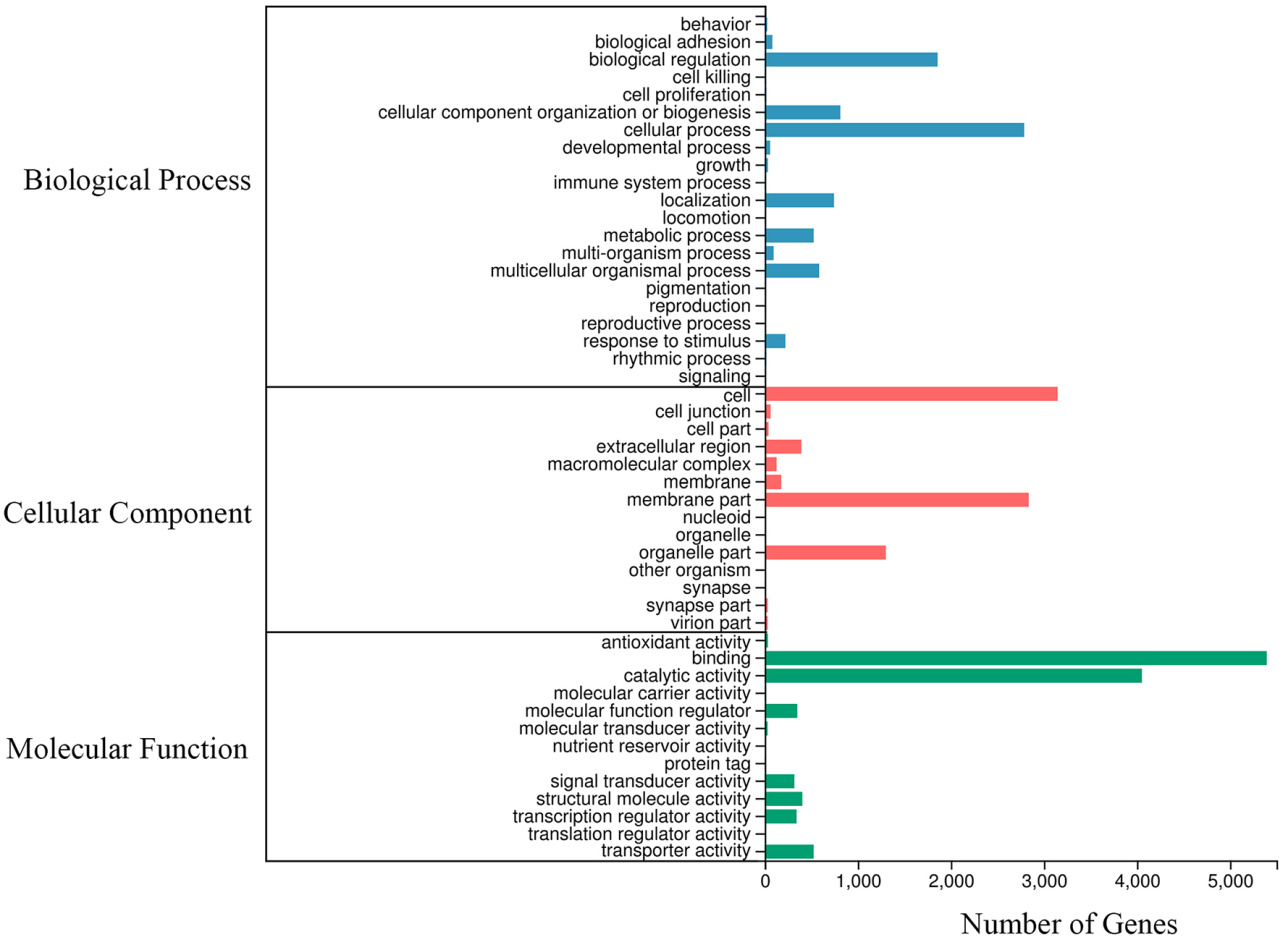
Clusters of GO categorizations of all annotated unigenes.

**Figure 4:**
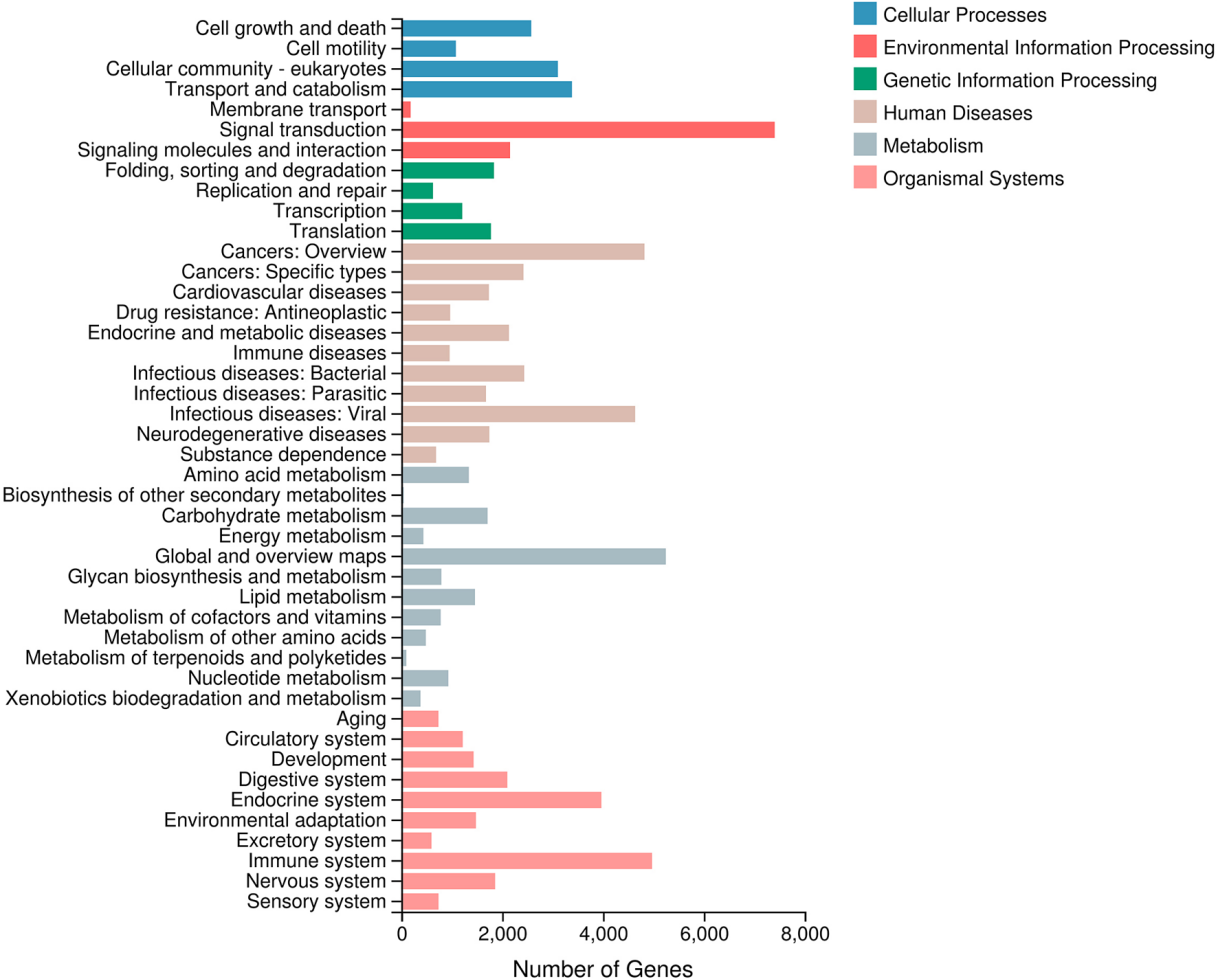
KEGG classifications of unigenes.

A total of 41,274 unigenes were annotated to the KOG database and classified into 25 subcategories. Among the 25 subcategories, ‘general function prediction only’ (6,963 unigenes) and ‘signal transduction mechanisms’ (5,992 unigenes) were the top two subcategories (Fig. 2). In addition, 11,056 unigenes were classified into three major GO categories (cellular component, biological process and molecular function) (Fig. 3). The terms ‘binding’ (5,395 unigenes), ‘catalytic activity’ (4,054 unigenes) and ‘cell’ (3,148 unigenes) were dominant in each functional category, respectively. In total, 46,302 unigenes were assigned to pathways based on the KEGG pathways analysis (Fig. 4); the top four pathways were ‘human diseases’ (24,232), ‘organism system’ (19,108), ‘metabolism’ (13,717) and ‘cellular processes’ (10,149). Furthermore, the predominant subcategories of pathways were ‘signal transduction’ (7406), ‘global and overview maps’ (5,246) and ‘immune system’ (4,972). These annotations offer a valuable resource for investigating specific functions, pathways and processes in teleost research.

### Discovery of molecular markers

**Figure 5:**
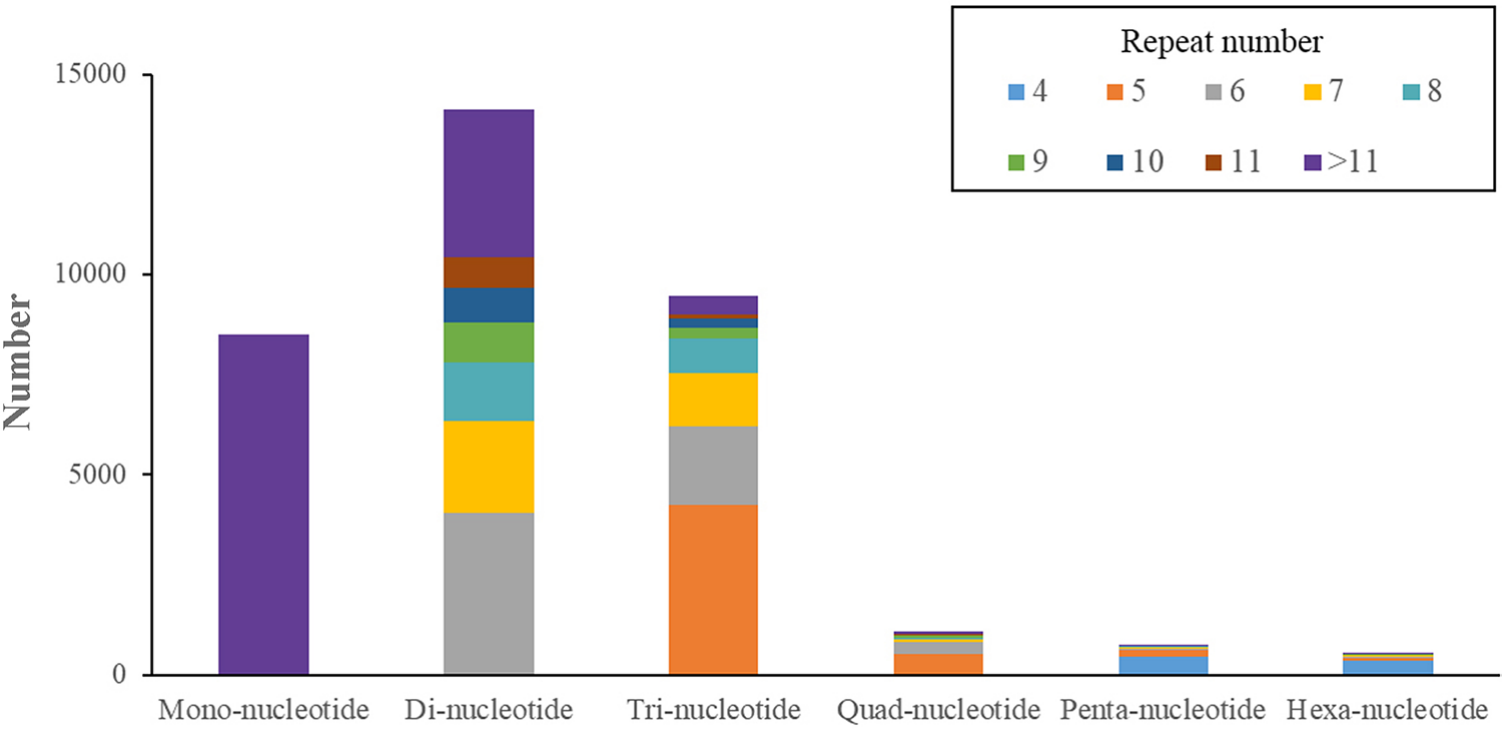
The distribution of different SSR.

SSRs have been widely applied in genetic linkage mapping, quantitative trait locus (QTL) mapping and population genetic diversity analyses for aquaculture species due to their high variability, abundance and co-dominant mode of inheritance^33^. Our search revealed 34,473 SSRs in the transcriptomic dataset, of which 41.00% were di-nucleotide repeats, 27.49% were tri-nucleotide repeats, and 24.66% were mono-nucleotide repeats (Fig. 5). Our findings are consistent with the conclusion that the di-nucleotide repeat type of SSR is the most abundant repeat type in animals^17,34^.

In the present study, fifty pairs of primers were randomly selected for synthesis and validation, and 36 primer pairs gave clean amplification product at the appropriate annealing temperatures. Among these, 19 primer pairs (38.00%) amplified at least four allele from all samples assessed on 6% denaturing polyacrylamide gels, indicating that these identified SSRs were at least moderately polymorphic (Table S1, Figure S2).

### Potential sex-related genes and functional enrichment analysis results

**Figure 6:**
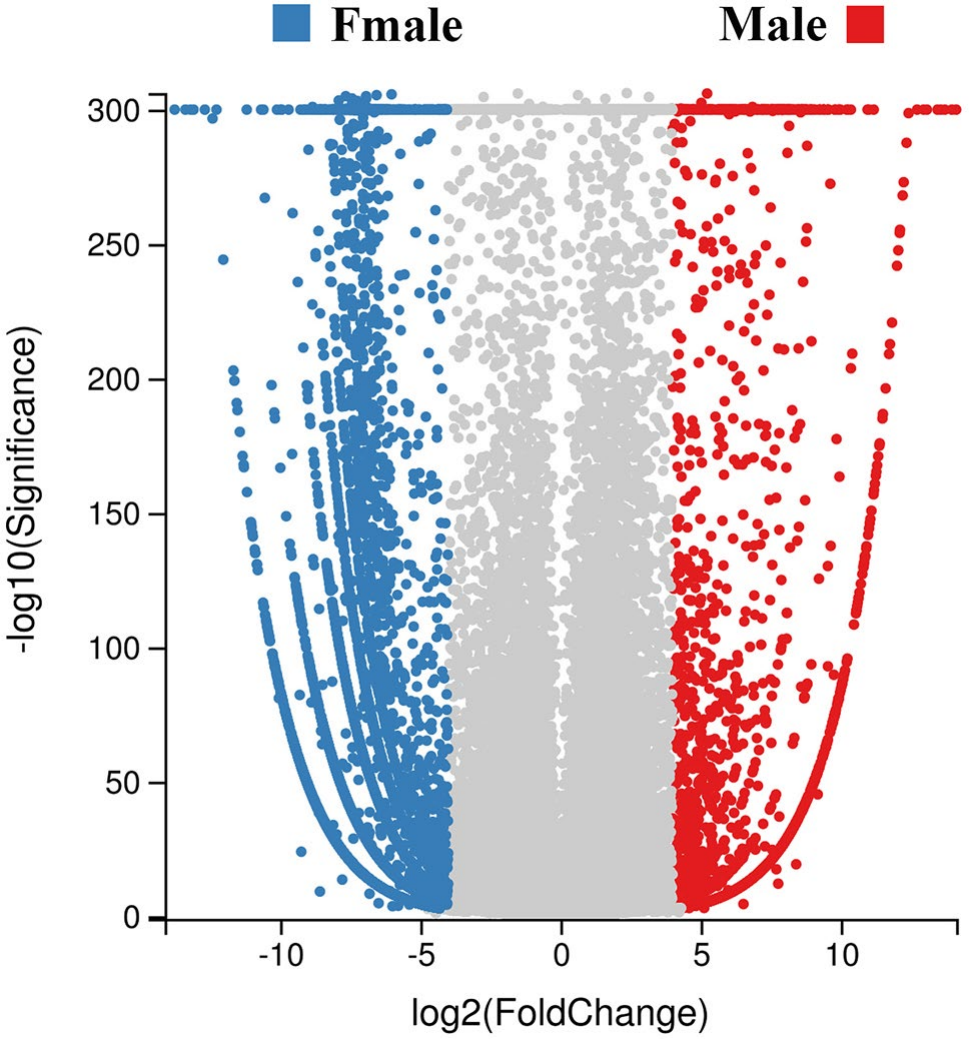
Volcano plot of the gene expression profiles from the libraries for the different sexes. Blue and red dots indicate female-based genes and male-biased genes, respectively.

To analyse the potential sex-related genes, unigenes with |log_2_(fold change)| values ≥ 4 and Q-values < 0.001 were determined to be DEGs between sexes. After filtration, 11,676 DEGs were obtained between different sexes of *P*. *edita*; these genes might be associated with sex determination (Fig. 6). Among these genes, 2,341 unigenes were male-biased DEGs and 9,335 unigenes were female-biased DEGs. In addition, sex-specific genes were detected, including 1,134 male SEGs and 5,463 female SEGs. All the DEGs between the two sexes were then used as input to perform GO and KEGG enrichment. Consequently, 2,543 unigenes (2,237 female-biased genes and 306 male-biased genes) were assigned to 1,911 GO terms (Figure S3). The results regarding the GO terms showed that sex-biased genes were predominantly associated with ‘integral component of membrane’ (GO:0016021), ‘ATP binding’ (GO:0005524), ‘nucleus’ (GO:0005634) and other terms. The top 20 statistically significant KEGG classifications of sex-biased genes are shown in Table S2. The results suggested that the three largest KEGG classifications of sex-biased genes were ‘Metabolic pathways’ (ko01100), ‘Epstein-Barr virus infection’ (ko05169) and ‘Small cell lung cancer’ (ko05200).

Sex determination is a complex trait that is controlled by multiple genes. Among the DEGs, genes involved in regulating sex control and gonadal development were predicted based on the results of sequence annotation, including genes encoding zona pellucida sperm-binding proteins (*zps*), anti-Müllerian hormone (*amh*), P450 aromatase (*cyp19a*), SRY-box containing protein 4 (*sox4*), and gonadal soma derived factor 1 (*gsdf*) and other candidate genes (Table 4).

**Table 4:** Identified sex-related genes in this study.

Gene ID	Fold change (♂/♀)	Q-value	Gene annotation
Unigene12885_All	6.806997266	3.15E − 27	SRY-box 4
Unigene30644_All	− 8.265581993	6.34E − 189	Zona pellucida sperm-binding protein 3-like
Unigene29033_All	− 8.189283619	0	Zona pellucida sperm-binding protein 4-like
CL829.Contig2_All	1.533826622	5.35E − 77	Gonadal soma derived factor
Unigene17197_All	− 4.963984212	0	P43 5S RNA-binding protein
CL169.Contig2_All	− 1.256662605	5.24E − 09	Vasa gene for ATP-dependent RNA helicase DDX4
Unigene14801_All	− 1.759691063	3.57E − 30	Meiotic nuclear divisions 1
Unigene14802_All	− 7.760008104	0	Zygote arrest 1
Unigene15812_All	− 5.058459684	0	Androgen receptor-like
Unigene19698_All	− 6.571625249	0	Wee1-like protein kinase 2
Unigene24074_All	− 3.198231905	7.21E − 231	Transcription factor IIIA-like
Unigene29297_All	− 8.157704591	0	Growth differentiation factor 9
Unigene29624_All	− 3.907248251	8.38E − 08	P450 aromatase
Unigene34643_All	1.260533072	2.70E − 04	Motile sperm domain containing 2
CL3719.Contig1_All	1.078840533	1.86E − 09	Nuclear transcription factor Y, alpha
Unigene12021_All	1.100442985	1.27E − 08	RAS-like estrogen regulated growth inhibitor
CL6709.Contig2_All	− 6.725894334	1.49E − 50	Gonadotropin II beta subunit
Unigene29732_All	− 1.907248	6.60E − 06	Anti-Muellerian hormone
CL3301.Contig1_All	1.192909262	1.72E − 41	Kinesin family member C3
Unigene26946_All	6.806997266	1.66E − 14	Steroidogenic acute regulatory protein

### Transcriptomic data validation

**Figure 7:**
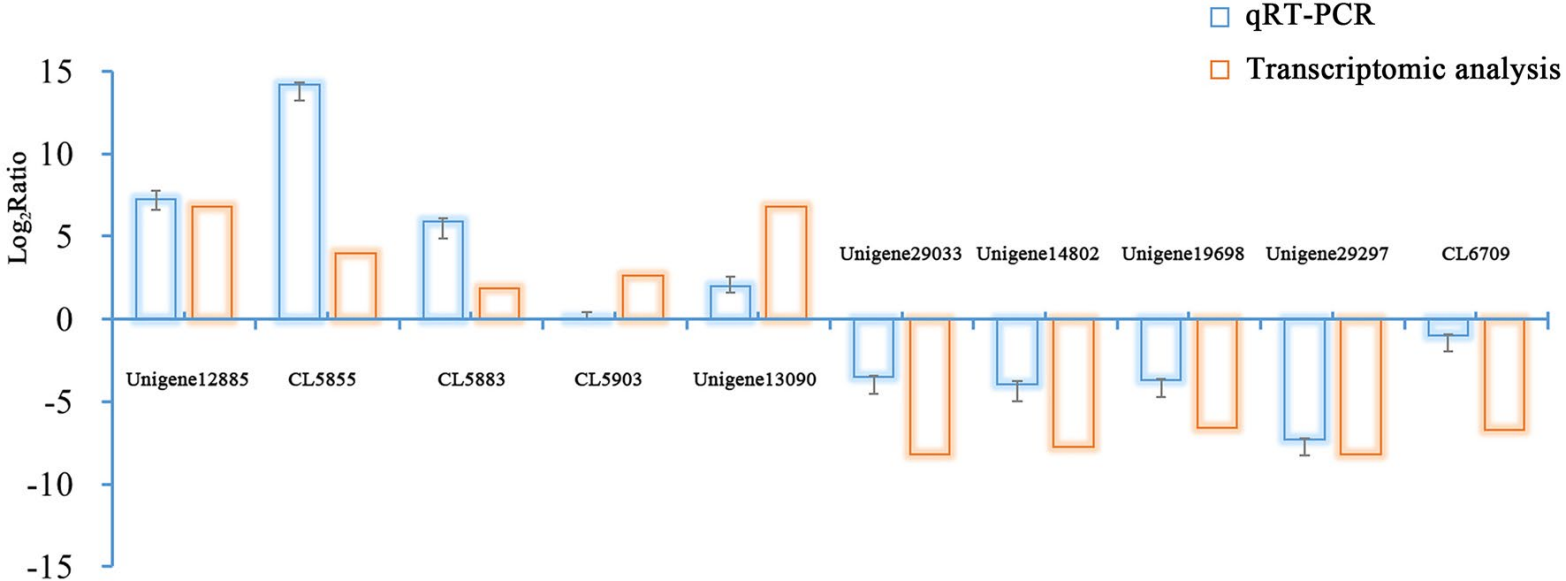
Expression levels of ten unigenes as determined by transcriptomic analysis and qRT-PCR.

To validate the transcriptomic data, ten selected genes (five female-biased genes and five male-biased genes) were subjected to qRT-PCR to investigate the expression profiles in all female and male samples. The specific primers are shown in Table S3. Similar up- or down-regulation patterns of these genes were observed in qRT-PCR and RNA-seq results, although few genes showed differentiation between the values (Fig. 7). These differentiations may be due to the different calculated methods used between RNA-seq and qRT-PCR. Thus, the results of qRT-PCR validated the transcriptomic data.

## Discussion

It is obvious that genes involved in gonad development and related to sex differentiation play critical roles in controlling the sex ratios of teleosts^17^. It is crucial to clarify the mechanisms of gonad differentiation and sex determination. However, due to insufficient detection of sex-related genes and explicit determination of biological pathways, research in this area has advanced very slowly. *Parargyrops edita*, an economically important marine fish, has rarely been studied with regard to its sex-related and sex-determining genes. To explore the mechanisms of sex determination, sex-related genes, and their biological pathways should be screened. In the present study, the transcriptomes of different sexes of *P*. *edita* were sequenced by the RNA-seq technique to screen sex-related genes between females and males. Our results detected numerous sequences that could be annotated to well-known sex-related genes and that were enriched in sex-related biological pathways. After transcriptome sequencing and assembly, 79,775 unigenes were obtained. After annotation, 54,255 (68.01%) unigenes were significantly matched in the protein databases. The annotation information of these unigenes might provide basic resources for further research on the molecular mechanisms of biological processes of *P*. *edita*. In this study, 11,676 DEGs were obtained between different sexes of *P*. *edita*. Some of these genes may play crucial roles in the sex determination of *P*. *edita*. Our results could provide fundamental resources for further studies on teleost.

According to the annotations, many sex-related genes were identified from the *P*. *edita* transcriptome. Zona pellucida proteins are a major class of female-specific glycoproteins related to reproduction in female vertebrates^35^. Zona pellucida proteins are detected in the mammalian egg envelope, and they are also in the inner layer of the fish chorion. Zona pellucida proteins are encoded by multiple genes, including *zp3*, *zp4* and potentially other zona pellucida proteins genes^36,37^. Recently, multiple copies of *zp* genes have been detected in some teleost fishes (e.g., medaka and carp)^38,39^. The zona pellucida sperm-binding protein 3 and zona pellucida sperm-binding protein 4 function as the primary sperm receptor and induces the acrosome reaction^35^. In this study, compared to male *P*. *edita*, female *P*. *edita* showed higher *zp3* and *zp4* expression levels, showing that these sex-specific genes may play critical roles in reproduction.

The sex-determining region Y (SRY) box, or SOX, is characterized as a conserved signature sequence in the high-mobility group (HMG) DNA-binding domain^40^. SOX genes have been identified in most vertebrates (mammals, birds and fish), and the orthologous genes in different species are highly similar^41^. It has been suggested that the SOX4 protein plays critical roles in many developmental processes, nervous system development, thymocyte development and embryonic cardiac development^42–45^. In this study, compared to female *P*. *edita*, male *P*. *edita* showed higher *sox4* expression levels.

Gonadotropins (GTHs) are important modulators of gonadal steroidogenesis and gametogenesis in teleosts, and GTH-II is important for final maturation and spermiation or ovulation^46,47^. In red seabream (*Pagrus major*), GTH-II may be involved in regulation of gametogenesis in both males and females and may be important for final maturation and ovulation or spermiation^48^. Tian et al. found that GTH-II subunit genes were expressed at higher levels in ovaries than in testes in *Sillago sihama*^22^. Similarly, GTH-II showed higher expression levels in female *P*. *edita* than in male *P. edita*, indicating that GTH-II may be important for final maturation and ovulation. Research has suggested that transforming growth factor beta (TGF-β) superfamily members play important roles in cellular homeostasis, cell growth and cell differentiation. An important member, anti-Müllerian hormone (*amh*) has been identified in some teleost fishes (e.g., *Oreochromis niloticus*, *Larimichthys crocea*, and *Cynoglossus semilaevis*); this gene plays a critical role in gonad development in several fish species^49,50^. Furthermore, another important member of the TGF-β family, gonadal soma-derived factor (*gsdf*), was first identified in *Oncorhynchus mykiss* and plays a critical role in the proliferation of spermatogonia in male rainbow trout^51^. However, it has been reported that *gsdf* is also expressed in the follicle cells surrounding oocytes in trout and salmon ^51,52^. In the present study, *gsdf* and *amh* showed higher expression levels in males and females, respectively, than in the opposite sexes indicating that these genes may mainly participate in the proliferation of spermatogonia and oogonia, respectively. Furthermore, the kinesin superfamily, a group of microtubule-dependent motors, has been suggested to play important roles in nuclear reshaping, acrosome biogenesis and flagellum formation during spermiogenesis^53,54^. KIFC3 is a C-terminal kinesin motor protein in the superfamily that is expressed mainly in cell lines and proliferative tissues^55^. It has been reported that KIFC3 genes play complementary roles in nuclear morphogenesis and acrosome formation during spermiogenesis of *Eumeces chinensis* ^56^. However, there is no evidence to support a correlation between KIFC3 genes and fish sex differentiation. Our study seems to indicate that KIFC3 genes are expressed mainly in male *P*. *edita*. The expression of KIFC3 genes should be studied in different organs of fish.

Doublesex and mab-3-related transcription factor 1 (*Dmrt1*), a sex-determining gene, has been identified in many vertebrate species^57–60^. *Dmrt1* is highly conserved and has been reported as the first conserved gene involved in sex differentiation found from invertebrates to humans^57^. In addition, *Dmrt1* has been indentified in many teleost fish, including *Sillago sihama*^21^, *Scophthalmus maximus*^6^, and *Cynoglossus semilaevis* ^61^. However, our data for *Dmrt1* was not consistent with data for this gene from other species. In addition, there were some other known sex-related genes whose expression was not detected in *P*. *edita*. This lack of detection could have occurred because the threshold for DEG determination was too high or too low or because these genes are not actually sex-regulators in *P*. *edita*.

In the present study, some enriched KEGG pathways related to sex determination were identified, including the ‘mitogen-activated protein kinase’ (MAPK) signaling pathway, the ‘neuroactive ligand-receptor interaction’ and the p53 signaling pathway.

The MAPK signaling pathway exists in all eukaryotic organisms and plays a wide variety of cellular roles in growth, differentiation, and stress responses^62^. Previous studies have shown that the MAPK signaling pathway mainly participates in the movement of primary spermatocytes across the blood-testis barrier and the regulation of gonadotropin subunit gene expression^63^. In the present study, the pathway was significantly enriched in male *P*. *edita*. It was also enriched in female *P*. *edita* but without statistical significance, which might indicate that the pathway plays different roles in the gonadal development of *P*. *edita*.

Previous studies have shown that the neuroactive ligand-receptor interaction signaling pathway is associated with lactation performance in mice. Furthermore, associated genes of the pathway are also upregulated^64^. In teleost fish, it has been suggested that this pathway plays important roles in teleost reproduction and gonadal development, such as in *Oplegnathus punctatus*^21^, *Paralichthys olivaceus*^20^, *Nile tilapia*^65^ and *Perca flavescens*^66^. Hence, we speculated that the pathway might serve as a critical modulator in both the reproductive system and the nervous system to control sex hormone production. Our results showed that the pathway might be correlated with gonadal development and sexual maturity in *P*. *edita* by regulating the production and neuro-distribution of sex steroids.

## Conclusions

In conclusion, this was the first transcriptomic study of *P*. *edita* and it provided significant information to enrich the genetic resources. Through comparison of male and female *P*. *edita* transcriptomes, numerous DEGs known to be involved in sex-related processes were identified. Through GO and KEGG enrichment analyses, several potential gonadal development- and gametogenesis-related genes and pathways were identified. These findings will be useful for clarifying the molecular mechanism of sex-determination and for future functional analyses of sex-associated genes. Moreover, many SSRs were identified in the transcriptome, providing tools for future molecular biology studies on this important species.

## Supplementary Information

**Supplementary Materials**: Figure S1: Completeness of the assembly and annotations, Figure S2: Polyacrylamide gel electrophoresis for 19 polymorphic SSR markers in 24 individuals, Figure S3: GO terms for the DEGs in the biological process, cellular component, and molecular function categories, Table S1: Characterization of 19 polymorphic SSR loci, Table S2: Top 20 KEGG pathways, Table S3: Specific primers for the selected unigenes and reference genes.
