# Supplementary material for: Comparative transcriptomic analysis for identification of candidate sex-related genes and pathways in Crimson seabream (Parargyrops edita)
Source: Sci Rep. 2021 Jan 13;11:1077. doi: 10.1038/s41598-020-80282-5 (PMC7806868; doi:10.1038/s41598-020-80282-5)
Supplement: Supplementary file 1 — Supplementary Materials: Figure S1: Completeness of the assembly and annotations, Figure S2: Polyacrylamide gel electrophoresis for 19 polymorphic SSR markers in 24 individuals, Figure S3: GO terms for the DEGs in the biological process, cellular component, and molecular function categories, Table S1: Characterization of 19 polymorphic SSR loci, Table S2: Top 20 KEGG pathways, Table S3: Specific primers for the selected unigenes and reference genes. [file 41598_2020_80282_MOESM1_ESM.docx]

**Comparative transcriptomic analysis for identification of candidate sex-related genes and pathways in Crimson** **seabream (*Parargyrops edita*)**

Binbin Shan, Yan Liu, Changping Yang, Yu Zhao and Dianrong Sun*

Key Laboratory of South China Sea Fishery Resources Exploitation & Utilization, Ministry of Agriculture Rural Affairs

Guangdong Provincial Key Laboratory of Fishery Ecology and Environment

South China Sea Fisheries Research Institute, Chinese Academy of Fisheries Sciences

***** Correspondence: [sundianrong@yeah.net](mailto:sundianrong@yeah.net); Tel.: 86-020-89100850


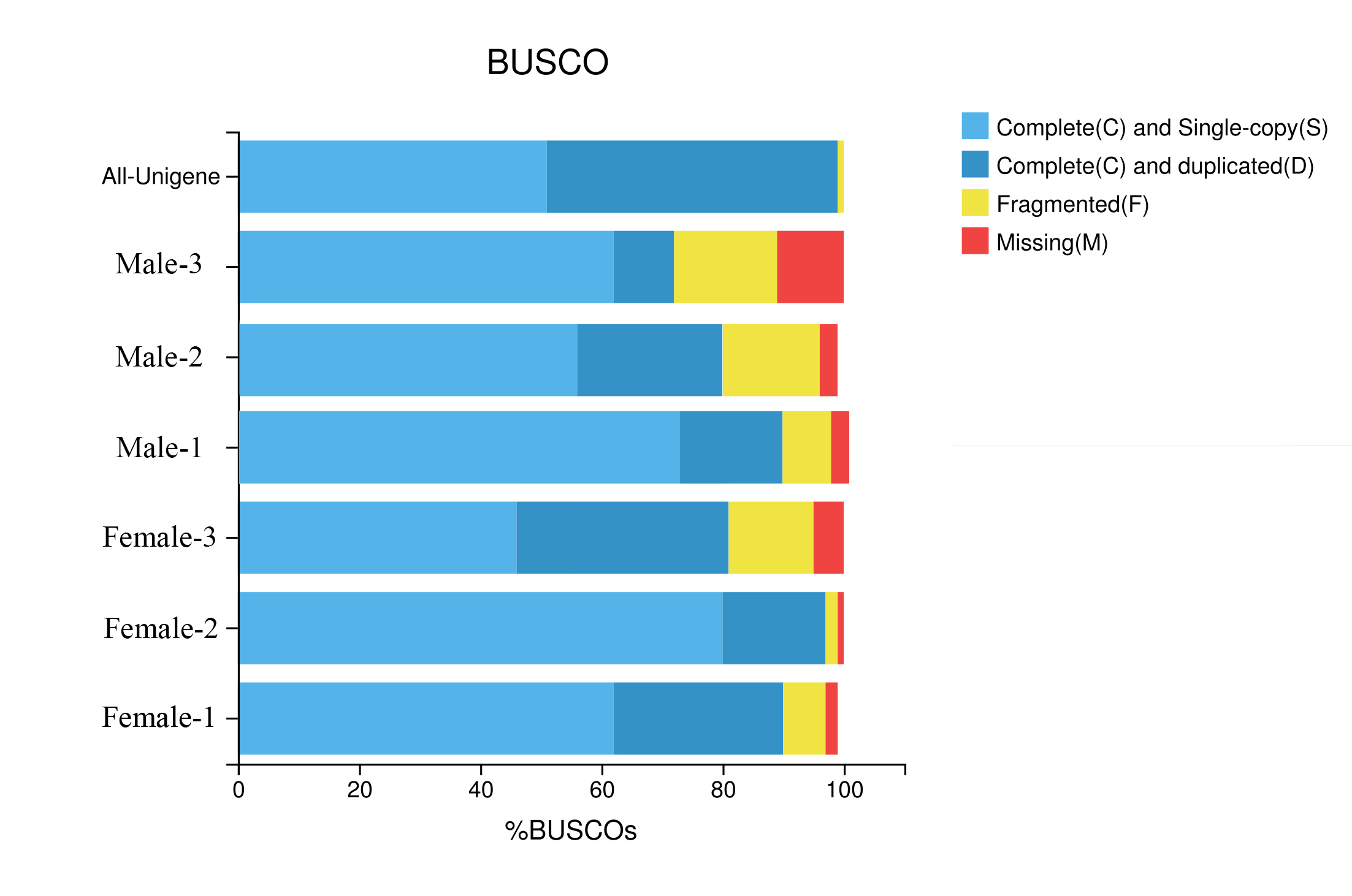


Figure S1. Completeness of the assembly and annotations.


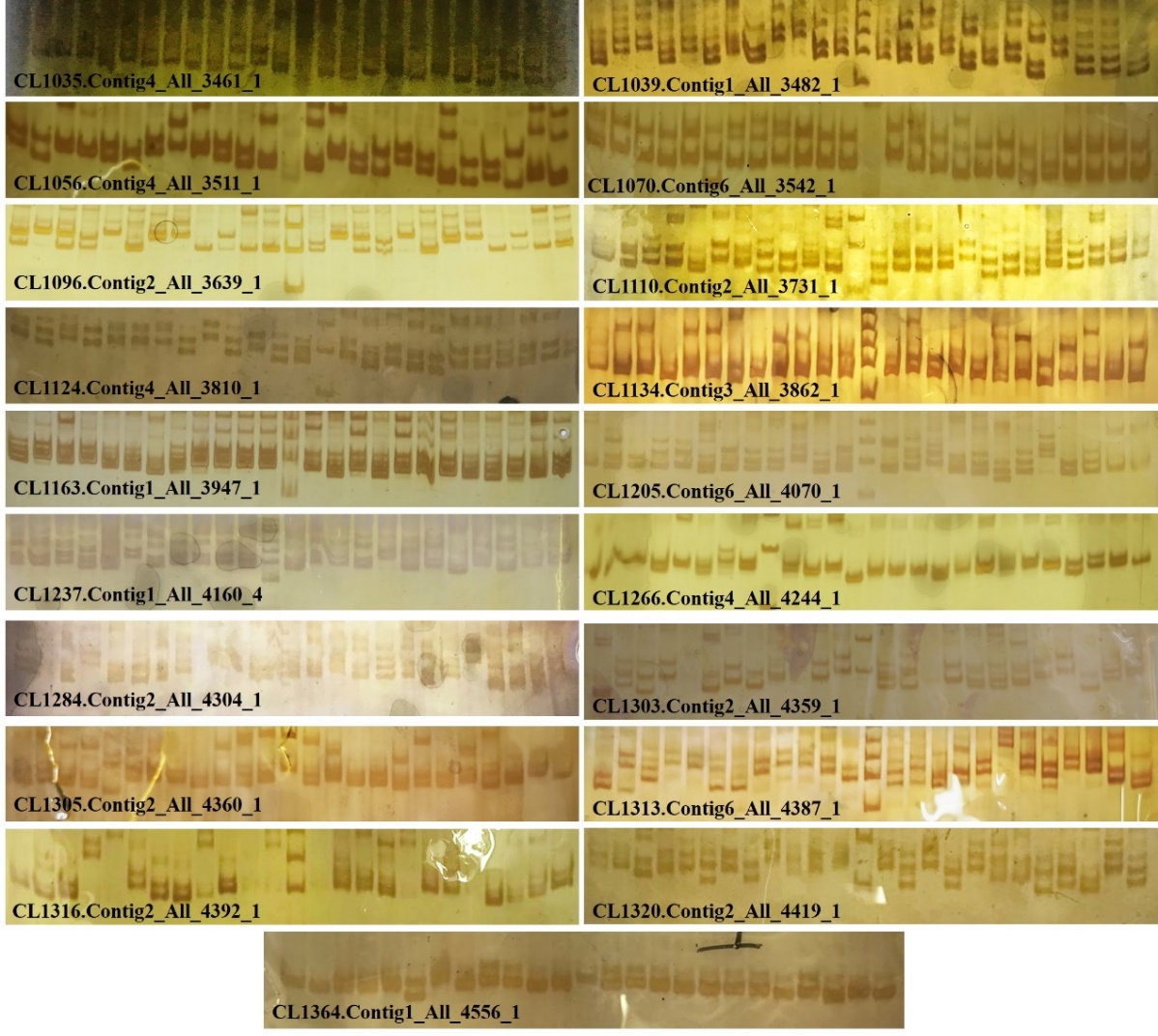


Figure S2. Polyacrylamide gel electrophoresis for 19 polymorphic SSR markers in 24 individuals.


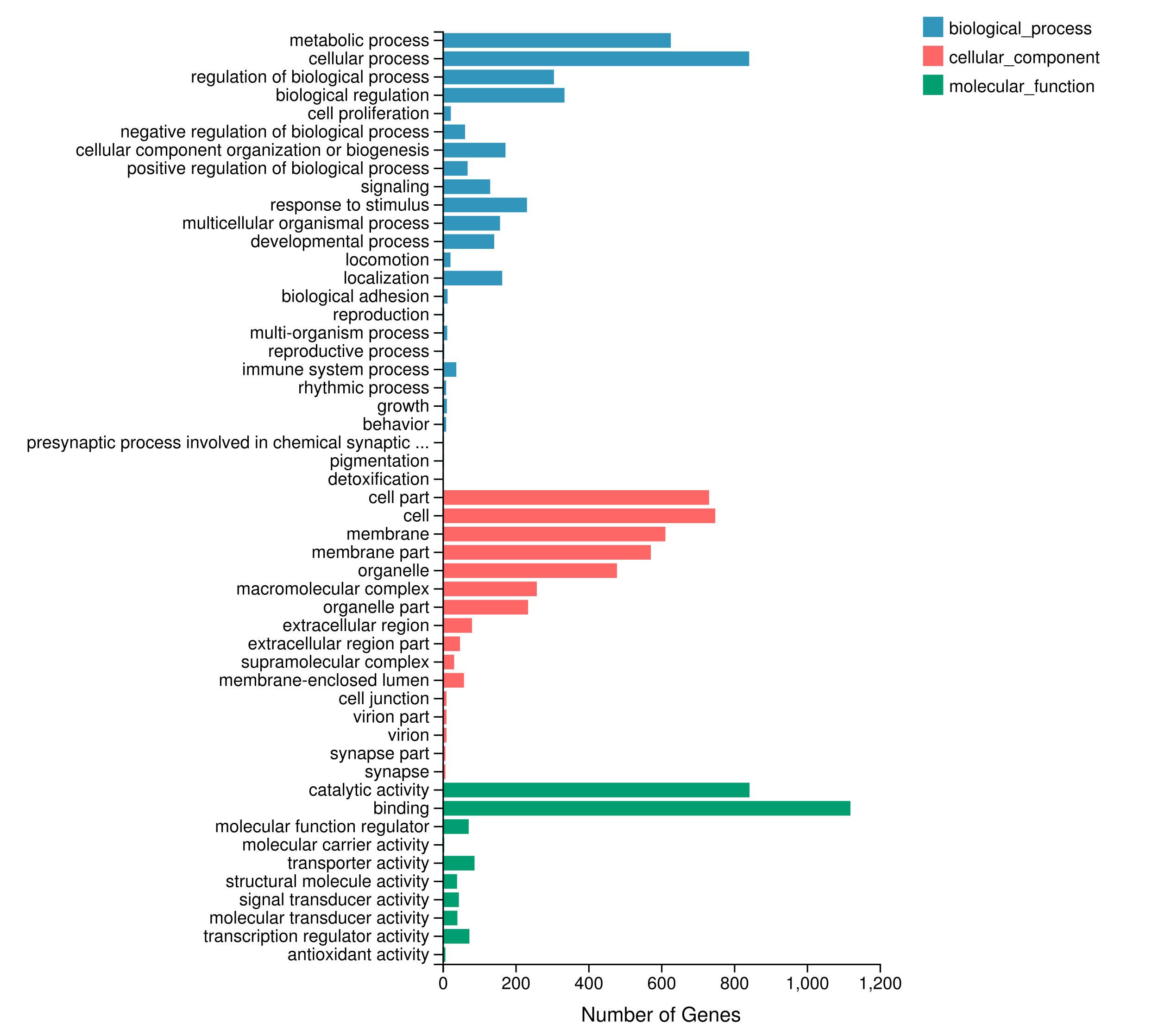


Figure S3. GO terms for the DEGs in the biological process, cellular component, and molecular function categories.

Table S1. Characterization of 19 polymorphic SSR loci.

| SSR id | Primer | Repeat unit | Temperature (°C) | Product size (bp) |
| --- | --- | --- | --- | --- |
| CL1035.Contig4_All_3461 | F-CCAGATGACAAGCAATCAAATAA | AC (2*18) | 59 | 159 |
|  | R-CAGCCATTTTGTTAGGTCCTG |  |  |  |
| CL1039.Contig1_All_3482 | F-GTTGCCGCTTGAAAAGAATCTA | AC (2*12) | 60 | 145 |
|  | R-ATATCCAAGTCAATTCCTCAGGC |  |  |  |
| CL1056.Contig4_All_3511 | F-GTCATCGTCTTTGACCTTCACTC | TCT (3*13) | 59 | 123 |
|  | R-CCCTGCCTTAAAGCATCATC |  |  |  |
| CL1070.Contig6_All_3542 | F-GCTGCAGTTTCTACTCAACACAA | TG (2*14) | 59 | 117 |
|  | R-AAAGAAAAACCTGTGGACACAAA |  |  |  |
| CL1096.Contig2_All_3639 | F-TCATCTGATACAAGACAACACGG | GAA (3*10) | 60 | 115 |
|  | R-GAAGAGGAGAAGGTTTGAGGTGT |  |  |  |
| CL1110.Contig2_All_3731 | F-CAGATGTTGTTCAGCTGAGAGG | TG (2*18) | 59 | 137 |
|  | R-TGCAGTAACTAACCCAGCTCTTC |  |  |  |
| CL1124.Contig4_All_3810 | F-AACTTCAGTCGCCTGTGTAAGAC | TG (2*26) | 60 | 160 |
|  | R-GTTGAGTGGTGATCGACATTTTT |  |  |  |
| CL1134.Contig3_All_3862 | F-AAAGACTAAAATTCACCAGTAACCA | ATA (3*13) | 59 | 142 |
|  | R-ACAGTGTGTTCTGCTGTGTCATT |  |  |  |
| CL1163.Contig1_All_3947 | F-TGGCAATCCTTTTTATTTCACAC | CA (2*14) | 60 | 145 |
|  | R-ACACTCACCCTCTCTTGTGTGTT |  |  |  |
| CL1205.Contig6_All_4070 | F-CTCTGTGGTTTCTGAGCTGTTCT | TG (2*19) | 60 | 153 |
|  | R-TTTCTCATGTTCGGTTTTAGCAC |  |  |  |
| CL1237.Contig1_All_4160_4 | F-ACAGGGACGTGATCTGTATGTTT | GA (2*15) | 60 | 96 |
|  | R-AGAGAAAGGATGTGTTGTGCAAT |  |  |  |
| CL1266.Contig4_All_4244 | F-AGAACAGGCACGTTGGAATACT | GT (2*28) | 59 | 146 |
|  | R-CGTGAAAGTGTTTTTCTGTGAGA |  |  |  |
| CL1284.Contig2_All_4304 | F-TTGTGTATTTGAGTGCTTGGTTG | CA (2*23) | 60 | 128 |
|  | R-GTCTGTCCACAGTTGGTTTGTCT |  |  |  |
| CL1303.Contig2_All_4359 | F-AAGGTTTGTGCGACTGATCTAAA | TG (2*10) | 60 | 127 |
|  | R-AACCCCATGTTTCACCATATGTA |  |  |  |
| CL1305.Contig2_All_4360 | F-AGCATGACTTTGGGATTTGTCTA | GT (2*14) | 62 | 97 |
|  | R-CTGTGCATCGAGAACAGCTACAC |  |  |  |
| CL1313.Contig6_All_4387 | F-TCCTGGTAAGACGACGTTAAAAA | GT (2*29) | 62 | 160 |
|  | R-TACTCAGCCACTTCCTCTTCACC |  |  |  |
| CL1316.Contig2_All_4392 | F-CAATTATATTGAGACCAACCCCA | AT (2*11) | 58 | 110 |
|  | R-AGACAAATTAAAAAGGCACAAGG |  |  |  |
| CL1320.Contig2_All_4419 | F-CCAAGTGACAGGTCCTTACAATC | AC (2*20) | 60 | 112 |
|  | R-AGAAGCACAGGGTGTAGGAGTTT |  |  |  |
| CL1364.Contig1_All_4556 | F-GAACGTGTGATAAAAGAACTGGG | TG (2*10) | 60 | 97 |
|  | R-CAACAAGAGATGGCCTGAATTAC |  |  |  |

Table S2. Top 20 KEGG pathways.

| Access number | Classifications | Unigenes number |
| --- | --- | --- |
| ko01100 | Metabolic pathways | 1025 |
| ko05169 | Epstein-Barr virus infection | 239 |
| ko05200 | Small cell lung cancer | 225 |
| ko04714 | Thermogenesis | 219 |
| ko05165 | Human papillomavirus infection | 211 |
| ko03040 | Spliceosome | 203 |
| ko05203 | Autoimmune thyroid | 200 |
| ko04530 | Tight junction | 195 |
| ko04141 | Protein processing in endoplasmic reticulum | 193 |
| ko05016 | Huntington's disease | 192 |
| ko05168 | Herpes simplex infection | 173 |
| ko04144 | Endocytosis | 172 |
| ko03013 | RNA transport | 168 |
| ko00230 | Purine metabolism | 168 |
| ko01200 | Carbon metabolism | 164 |
| ko04151 | PI3K-Akt signaling pathway | 162 |
| ko04510 | Hypertrophic | 152 |
| ko05205 | Proteoglycans in cancer | 149 |
| ko05166 | HTLV-I infection | 145 |
| ko04010 | MAPK signaling pathway | 143 |

Table S3. Specific primers for the selected unigenes and reference genes.

| Primers | Sequences (5'to3') |
| --- | --- |
| 187-1>Unigene12885-F | AAAGCAGAGTGCGTGGCATC |
| 187-1>Unigene12885-R | TCTCTCCACTTTCCATGCGG |
| 187-2>Unigene29033-F | GGTGACATCCGCAGACAACC |
| 187-2>Unigene29033-R | CCCAACACCTTCCCTTGGAC |
| 187-3>Unigene14802-F | TGGTGGTTGTTGAAGGCCTC |
| 187-3>Unigene14802-R | ATCTTACGGCCCCTACACGG |
| 187-4>Unigene19698-F | CTCATGGATCGCGCAGTCTT |
| 187-4>Unigene19698-R | TGTCACCCAAGCAGAGCCTC |
| 187-5>Unigene29297-F | ACCGTAGATGAAGCCCATGG |
| 187-5>Unigene29297-R | TGCGATTTAGCCAGCTCAAA |
| 187-6>CL6709-F | TGCGTGCTGACTCACTGACA |
| 187-6>CL6709-R | TCCAGGATGAAACGTGCTCA |
| 187-8>CL5855-F | GCGTTTTATCAAAGCAAACA |
| 187-8>CL5855-R | TTCGAGACAGCGAGTAAACA |
| 187-9>CL5883-F | GCAACAGCAGCTTGAGAAGA |
| 187-9>CL5883-R | CGGAATGACGTGACATCCTA |
| 187-10>CL5903-F | GACCCTTGTTGGCTCGCTTC |
| 187-10>CL5903-R | AGTGCCCAGCGCATACACTG |
| 187-11>β-actin-F | ATCGTTCGTGACATTAAGGA |
| 187-11>β-actin-R | CAAGGAATGAAGGCTGGAA |
| 187-12>18S-F | GAAGGATTGACAGATTGAGAG |
| 187-12>18S-R | GTAGCGACGGACACATAT |
| 187-13>Unigene13090-F | TGTTACCTGCTCCGCTCTGG |
| 187-13>Unigene13090-R | CGTGCAGTCCTCGTGGATCT |
